# On the Use of OPEFB-Derived Microcrystalline Cellulose and Nano-Bentonite for Development of Thermoplastic Starch Hybrid Bio-Composites with Improved Performance

**DOI:** 10.3390/polym13060897

**Published:** 2021-03-15

**Authors:** Di Sheng Lai, Azlin Fazlina Osman, Sinar Arzuria Adnan, Ismail Ibrahim, Awad A. Alrashdi, Midhat Nabil Ahmad Salimi, Anwar Ul-Hamid

**Affiliations:** 1 Faculty of Chemical Engineering Technology, University Malaysia Perlis (UniMAP), Arau 02600, Malaysia; ericson_1206@hotmail.com (D.S.L.); sinar@unimap.edu.my (S.A.A.); ismailibrahim@unimap.edu.my (I.I.); nabil@unimap.edu.my (M.N.A.S.); 2 Biomedical and Nanotechnology Research Group, Center of Excellence Geopolymer and Green Technology (CEGeoGTech), Universiti Malaysia Perlis (UniMAP), Arau 02600, Malaysia; 3 Chemistry Department, Umm Al-Qura University, Al-Qunfudah University College, Al-qunfudah Center for Scientific Research (QCSR), Al Qunfudah 21962, Saudi Arabia; 4 Core Research Facilities, King Fahd University of Petroleum & Minerals, Dhahran 31261, Saudi Arabia; anwar@kfupm.edu.sa

**Keywords:** oil palm empty fruit bunch (OPEFB), microcrystalline cellulose, nano-bentonite, bio-composites, tensile properties

## Abstract

Thermoplastic starch (TPS) hybrid bio-composite films containing microcrystalline cellulose (C) and nano-bentonite (B) as hybrid fillers were studied to replace the conventional non-degradable plastic in packaging applications. Raw oil palm empty fruit bunch (OPEFB) was subjected to chemical treatment and acid hydrolysis to obtain C filler. B filler was ultra-sonicated for better dispersion in the TPS films to improve the filler–matrix interactions. The morphology and structure of fillers were characterized by scanning electron microscope (SEM), Fourier transform infrared spectroscopy (FTIR) and X-ray diffraction (XRD). TPS hybrid bio-composite films were produced by the casting method with different ratios of B and C fillers. The best ratio of B/C was determined through the data of the tensile test. FTIR analysis proved the molecular interactions between the TPS and the hybrid fillers due to the presence of polar groups in their structure. XRD analysis confirmed the intercalation of the TPS chains between the B inter-platelets as a result of well-developed interactions between the TPS and hybrid fillers. SEM images suggested that more plastic deformation occurred in the fractured surface of the TPS hybrid bio-composite film due to the higher degree of stretching after being subjected to tensile loading. Overall, the results indicate that incorporating the hybrid B/C fillers could tremendously improve the mechanical properties of the films. The best ratio of B/C in the TPS was found to be 4:1, in which the tensile strength (8.52MPa), Young’s modulus (42.0 MPa), elongation at break (116.4%) and tensile toughness of the film were increased by 92%, 146%, 156% and 338%, respectively. The significantly improved strength, modulus, flexibility and toughness of the film indicate the benefits of using the hybrid fillers, since these features are useful for the development of sustainable flexible packaging film.

## 1. Introduction

Conventional plastics bring so much convenience to our life. We consume plastics in our day-to-day life and in many applications, including food packaging and drinking straws [1]. Conventional plastic or fossil-fuel plastic has many unique characteristics that make it one of the most essential materials in manufacturing a product, such as having a wide range of processing temperatures, high chemical resistance, a high strength to weight ratio, ease of processing and low costs [2]. These properties indicate the versatility of plastic materials, and therefore they are being used to substitute other materials such as metal and ceramic in certain applications. Unfortunately, plastic’s great properties are sometimes overshadowed by its non-degradable properties which cause disposal problems and pollution on earth [3]. Nowadays, the plastic disposal problem is getting more severe. Thus, researchers are focusing on producing bio-plastics from natural sources such as starch which can be obtained from rice, corn, potato, and pea to solve the problem highlighted above.

Among all the biopolymers, starch has drawn significant attention from the researchers owing to its unique characteristics. Starch can be processed using the same processing techniques as conventional plastic, such as injection, extrusion and thermoforming. Starch is biodegradable and can be plasticized into a more useful form of plastic, which is called thermoplastic starch (TPS) [4]. However, to compete with conventional plastic, TPS-based plastics should possess comparable mechanical properties and barrier properties to conventional plastic. Yet, this is very difficult to achieve since thermoplastic starch inherited hydrophilic properties; thus, it has high sensitivity toward moisture absorption from the surroundings. The mechanical properties of thermoplastic starch decrease exponentially with the increase in humidity [5]. In order to improve the mechanical and barrier properties of thermoplastic starch, inorganic fillers or organic fillers are added to thermoplastic starch to increase its mechanical strength and barrier properties [6].

Palm oil is considered as one of the most important edible oils in the world, next only to soybean. However, palm oil extraction will generate an enormous amount of palm oil biomass wastes that consume up a large landfill area and endangering the environment [6]. Oil palm empty fruit bunch (OPEFB), as one of the major palm oil biomass wastes, contains a high percentage of cellulose of about 40–60%, which is the highest compared to kenaf, corn, and bagasse fiber [7]. Therefore, extraction of microcrystalline cellulose from the OPEFB is one of the effective strategies to add value the waste material.

In the search of new fillers for TPS that capable of enhancing its properties for various applications, the idea of using hybrid fillers (more than one type of filler) arrived due to the demands of consumers for biodegradable plastic packaging with combination of strength and toughness properties. The use of hybrid fillers may allow more comprehensive improvement in the properties of the TPS film by providing synergistic effect through the combination of both filler’s properties [8].

In this study, we have investigated the efficiency of using hybrid nano-bentonite (in-organic nano-clay) and OPEFB derived microcrystalline cellulose (organic fiber) fillers in improving the mechanical properties of the TPS bio-composite plastic film, particularly its tensile strength and flexibility (toughness). The microcrystalline cellulose and ultra-sonicated nano-bentonite were expected to have good interaction with the TPS due to their hydrophilic properties, thus enhancement in the TPS film’s mechanical properties could be gained. However, the TPS bio-composite film’s optimal strength can only be achieved when the nano-bentonite and microcrystalline cellulose are well dispersed in the matrix of the biopolymer. Nonetheless, nano-bentonite particles always tend to agglomerate when dispersed in the polymer matrix. To encounter this, ultra-sonication treatment is applied to improve dispersion of the nano-clay filler in a polymer matrix [6].

Tensile toughness property of a material relates to its capability to possess both high tensile strength and high flexibility when stretched. So far, there have been no published papers reporting an increase in both the strength and toughness of the bio-polymeric film with the addition of fillers; in fact, most articles report a decrease in the flexibility of the film along with the increase in its strength [9,10,11,12,13,14]. This is because high-modulus filler can restrict the molecular motions of the biopolymer matrices, lowering its elongation at break value and thus making it more brittle. Unfortunately, this characteristic is not suitable for flexible film packaging applications because the ability to stretch without breaking (tough) is a key requirement of such a film. Therefore, there is the need to discover new hybrid fillers that are capable of improving the toughness property of the bio-composite film by elevating both tensile strength and elongation at break values of the material. In this work, we have proved that enhancement in both tensile strength and elongation at break can be gained with the use of B/C hybrid fillers. As far as the bio-composite with a dual-type filler system (hybrid) is concerned, this discovery is the first to be reported. Furthermore, the dual hybrid filler system which comprised the OPEFB-derived microcrystalline cellulose and nano-bentonite was first to be introduced in the TPS bio-composite system. Both fillers were characterized by scanning electron microscope (SEM), X-ray diffraction (XRD) and Fourier transform infrared spectroscopy (FTIR). Bio-composite films with several ratios of B/C filler were produced using the casting technique. A tensile test was employed to determine the TPS bio-composite films’ mechanical properties containing a different ratio of microcrystalline cellulose and nano-bentonite. The optimal ratio of the OPEFB microcrystalline cellulose/nano-bentonite of the hybrid fillers that was capable of providing the best reinforcing and toughening effects to the TPS bio-composite film was determined. 

## 2. Materials and Methods

### 2.1. Materials 

The starch granules and nano-bentonite were supplied by Sigma-Aldrich (St. Louise, MO, USA). It was supplied by Euroscience Sdn. Bhd (Kuala Lumpur, Malaysia). The glycerine, sodium chlorite (NaClO_2_) and sodium bicarbonate were supplied by HmbG Chemicals (Hamburg, Germany) purchased through A.R Alatan Sains Sdn Bhd (Alor setar, Malaysia). Sulfuric acid was supplied by Fisher Chemical^TM^ (Concord, NH, USA) purchased through A.R Alatan Sains Sdn Bhd (Alor setar, Malaysia). OPEFB fiber was purchased from United Oil Palm Industries Sdn Bhd (Nibong Tebal, Malaysia). Deionized water was used as a solvent for all the experimental works.

### 2.2. Preparation of Treated OPEFB Cellulose from Raw OPEFB

The chemical isolation processes of the OPEFB were in accordance with the one reported by Zailuddin et al. [15]. The OPEFB was treated with 4% of NaOH solution at a temperature of 80 °C with constant stirring for one hour. The treated filler was washed with distilled water and filtered several times to ensure the removal of alkaline. This process was repeated four times to totally remove the hemicellulose. After that, the treated OPEFB fiber underwent bleaching treatment using dilute sodium chlorite (NaClO_2_) solution at a temperature of 80 °C under stirring conditions for 1 h. The treated fiber was washed with distilled water and filtered for several times to ensure the removal of NaClO_2_. These bleaching and washing procedures were repeated three times to ensure the removal of lignin and hemicellulose. The resultant cellulose is called treated OPEFB cellulose.

### 2.3. Preparation of Microcrystalline Cellulose (C) from the Treated OPEFB Cellulose

The treated OPEFB cellulose was subjected to concentrated acid hydrolysis with 64% H_2_SO_4_ at 45 °C for 1 h under stirring conditions. The suspension was added with 5-fold of cold water to stop the reaction. The cellulose suspension was centrifuged for 15 min at the speed of 7500 rpm and washed with distilled water to remove the acid content. This procedure was repeated at least ten times or until the pH of the solutions reached the value of 7 measured by using a pH meter. The produced microcrystalline cellulose was homogenized at 5000 rpm for 30 s and dried in the oven at 45 °C for 24 h.

### 2.4. Plasticization of Starch to Thermoplastic Starch (TPS)

Five grams of corn starch powder was added with 100 mL water into 250 mL beaker. After that, 2 g of glycerine was added into the starch solution to plasticize the starch. Then, the mixture was continuously stirred using a magnetic stirrer (500 rpm) for 45 min in a water bath, in which the temperature was maintained to be around 75–85 °C. A gel-like suspension of starch was obtained, showing that the starch has been transformed into the TPS.

### 2.5. Ultra-Sonication of Nano-Bentonite (B)

Nano-bentonite (B) was dispersed in deionized water in the ratio 1:20 and kept stirring under constant speed with a magnetic stirrer at room temperature. The (B) suspension was ultra-sonicated with an ultrasonic probe for 120 min with 20 s pulse on and 10s pulse off under 20% amplitude by using Branson Digital Ultrasonic Disruptor, Model 450D supplied by ProSciTech (Queensland, Australia). The suspension was filtered and dried in the oven. The dried powder was ground and sieved through a 53-micron sieve for de-agglomeration.

### 2.6. Preparation of TPS/B and TPS/C Bio-Composite and TPS/B/C Hybrid Bio-Composite Films

The hybrid fillers of microcrystalline cellulose (C) and nano-bentonite (B) were added into the TPS solution and continuously stirred at 75–85 °C. After that, 0.5 g of sodium bicarbonate was added into 20 mL water in a 50mL beaker and then added into the TPS mixture. The mixture was continuously stirred for about 5 min to obtain viscous and transparent suspension. Then, the mixture was poured into Teflon-coated mold and placed in the oven at 45 °C for 24 h for drying. The unfilled TPS, TPS/B and TPS/C bio-composite films were also produced as control samples. The formulation of all samples and acronyms is summarized in Table 1.

### 2.7. Fourier Transform Infrared Spectroscopy (FTIR) 

The FTIR analysis of the raw OPEFB, OPEFB-treated cellulose, OPEFB microcrystalline cellulose and nano-bentonite was performed by using the KBr pellet method. The functional groups of all the samples were analyzed using a Perkin Elmer spectrum 65 FTIR spectrometer (Waltham, MA, USA) with the wavenumber range of 650–4500 cm^−1^, 16 scans and resolution of 4 cm^−1^. FTIR transmitted infrared radiation through the sample and resulted in the infrared spectrum of the sample.

### 2.8. Scanning Electron Microscope (SEM)

The surface structure and morphology of fillers were analyzed using scanning electron microscope (SEM) (JEOL JSM-6460LA) (JEOL. Ltd., Tokyo, Japan). The morphology of raw OPEFB fiber, treated OPEFB cellulose and microcrystalline cellulose was studied under SEM to allow for comparison. Before images were captured, the fillers were coated with platinum using JFC-1600 Auto Fine Coater (JEOL Ltd., Tokyo, Japan) for getting the optimal SEM image of fillers. The surface and morphology of the raw OPEFB fiber and the treated OPEFB cellulose samples were taken at a magnification of ×500 and ×4000. Meanwhile, the morphologies of the microcrystalline cellulose and the ultra-sonicated nano-bentonite were taken at a magnification of ·30k and ·10k, respectively.

### 2.9. X-ray Diffraction (XRD) 

Raw OPEFB, OPEFB cellulose and OPEFB microcrystalline cellulose were analyzed by using a Bruker D2 Phaser X-Ray diffractometer (Billerica, MA, USA) using Cu Kα X-rays. The samples were tested by using a scan rate of 0.1 s per step from 2θ = 10–40°. The crystallinity index (CrI) of all samples was calculated according to the following equation:

(1) $$\mathrm{Crystallinity}\;\mathrm{Index}\;(\mathrm{CrI})\;=\;\frac{I-I’}{I}\;\times \;100$$

where: I = the height of the intensity for the crystalline peak measured at 2θ = 22–23°.; I’= the height of the intensity for the amorphous peak measured at 2θ = 18–19°.

The basal spacing and crystallinity of the nano-bentonite (before and after ultra-sonication) were analyzed using a Bruker D2 Phaser X-ray diffractometer with Cu Kα X-rays. The samples were tested using a scan rate of 0.1 s per step from 2θ = 5–40°. The XRD data were analyzed by using high score plus software. The basal spacing of the nano-bentonite was calculated by using Bragg’s Law (nλ = 2d sin θ).

### 2.10. Tensile Test

The tensile tests of the TPS, TPS bio-composite and TPS hybrid bio-composite films were performed by Instron machine model-5582 (Norwood, MA, USA),according to ASTM D 638 Type V. The samples were tested with a crosshead speed of 10 mm/min to obtain the stress–strain curves. From the data, the tensile properties, such as tensile strength, Young’s modulus, elongation at break and tensile toughness, were determined.

## 3. Results and Discussion

### 3.1. Morphological Characterization of Raw OPEFB Fiber, Treated cellulose, Microcrystalline Cellulose and Nano-Bentonite

**Figure 1:**
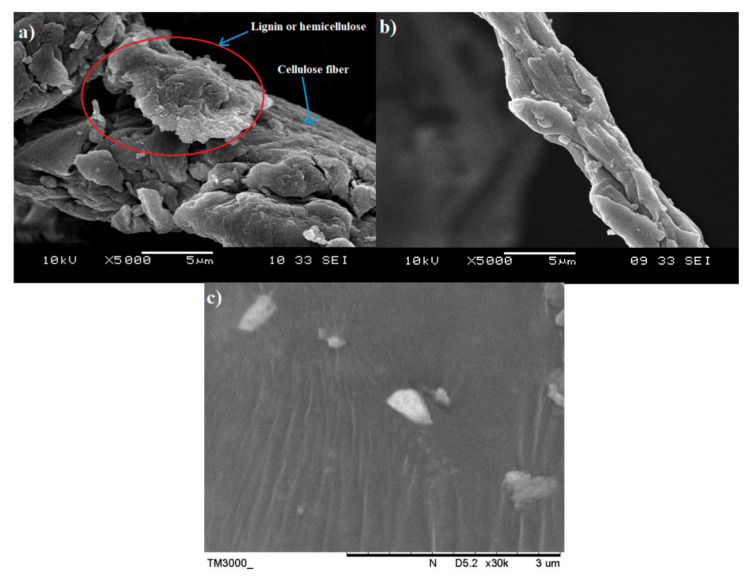
Scanning electron microscope (SEM) micrographs of: raw oil palm empty fruit bunch (OPEFB) fiber taken at ×5000 (**a**); treated OPEFB cellulose taken at ×5000 (**b**); microcrystalline cellulose taken at ×30k (**c**).

SEM analysis was performed to have a better understanding of the microstructural changes of the OPEFB caused by the pre-treatment and acid hydrolysis process. Figure 1a shows the surface morphology of the raw OPEFB fiber, while Figure 1b presents the structure of the OPEFB cellulose (after it underwent alkaline pre-treatment and the bleaching process). Apparently, there are changes in the OPEFB morphology detected through the comparison of both micrographs indicating the success of the chemical pre-treatment procedure in removing non-cellulosic materials such as hemicellulose, lignin and waxy substances from the raw OPEFB fiber.

The raw OPEFB fiber in Figure 1a possesses an irregular shape and the surface of the microfibrils cellulose (pointed by the blue arrow) was covered up by non-cellulosic materials which act as the protective surface (as marked by red circles). In contrast, Figure 1b shows the smooth, clear and individualized rod-like microfibrils surface of cellulose. The different morphology of the surface structure in the raw OPEFB (Figure 1a) and the treated OPEFB cellulose (Figure 1b) indicates that chemical pretreatment has successfully removed the non-cellulosic materials such as hemicellulose, lignin and waxy substances from the raw OPEFB fiber, which is in line with the FTIR results. Due to these removals, the microfibrils surface of the cellulose has been exposed and the crystallinity region of the cellulose was increased, as confirmed through the XRD analysis. Figure 1c represents the morphology of the microcrystalline cellulose after being dried up and observed under SEM with ×30k magnification. It can be observed that the structure of the OPEFB cellulose’s microfibril was completely destroyed and the size of the cellulose was significantly being reduced. However, the SEM diagram suggests that the microcrystalline cellulose tends to aggregate together into large particles of cellulose. Agglomeration of cellulose usually occurs after being dried from the suspension form [15].

**Figure 2:**
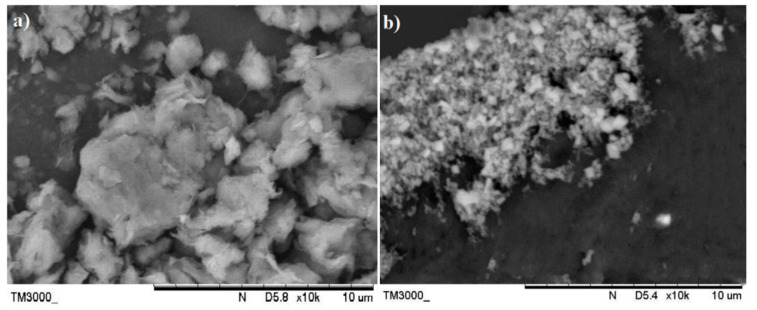
SEM picture of: nano-bentonite (before ultrasonication) (**a**); nano-bentonite (after ultrasonication) (**b**), taken at ×10k.

Figure 2a,b display the SEM images of the pristine nano-bentonite and ultra-sonicated bentonite, respectively. By comparing both figures, we can prove that the particle size of the nano-bentonite had been reduced significantly upon the ultra-sonication process. Before ultra-sonication, the nano-bentonite appears in large particles with an irregular shape and size. This was due to the high stacking of platelets forming large tactoids of the phyllo-silicate structure. After being ultra-sonicated, the particle size of the nano-bentonite was seen to greatly reduce, with rougher surface morphology. This observation was due to the tactoid size reduction of the nano-clay as a result of the delamination of the nano-platelets. However, in agglomerated form, it is difficult to get a clear image of the individual platelet of the nano-bentonite and determine its particle size. However, the results can confirm that the ultra-sonication process has successfully reduced the tactoid size of the nano-bentonite.

### 3.2. Chemical Structure of Raw OPEFB Fiber, Treated Cellulose, Microcrystalline Cellulose and Nano-Bentonite

**Figure 3:**
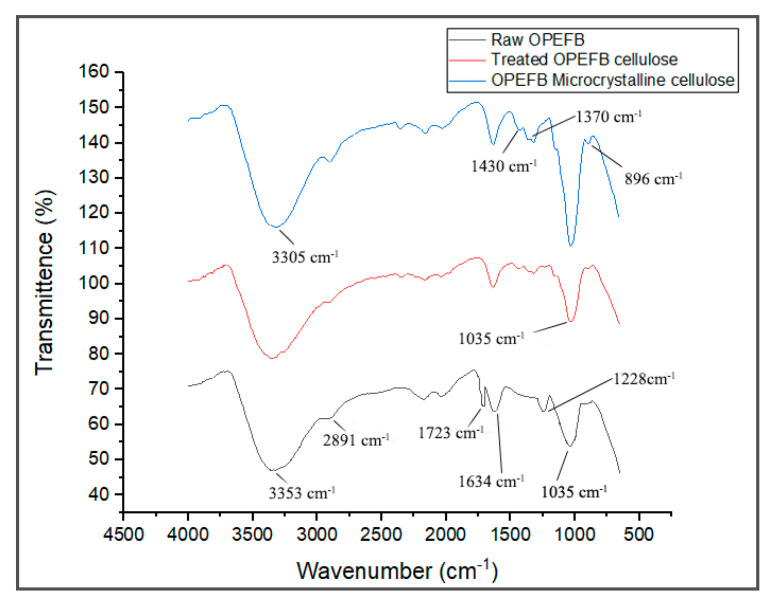
Fourier transform infrared spectroscopy (FTIR) spectra of raw OPEFB fiber, treated OPEFB cellulose fiber and OPEFB microcrystalline cellulose.

FTIR spectroscopy is a non-destructive method to study the chemical composition of material by analyzing its functional groups. The FTIR spectra of the raw OPEFB fiber, treated OPEFB cellulose and OPEFB microcrystalline cellulose are illustrated in Figure 3. Generally, the FTIR spectra of all samples were almost the same, indicating that they possess the same chemical composition and structure.

The FTIR spectra of the raw OPEFB and treated OPEFB cellulose represent a broad peak at the region of 3353 and 2891 cm^−1^ due to the –OH stretching of the aliphatic structure and aliphatic saturated C-H stretching of CH_2_ and CH_3_. The 3353 cm^−1^ can also be attributed to moisture content where the hydroxyl group is found in cellulose, hemicellulose and lignin. This shows that the cellulose is hydrophilic in nature [16]. The peak at 1035 cm^−1^ appears in both samples and represents the characteristics of anhydroglucose chains with the C-O-C stretch [17]. The treated cellulose has a sharper peak than the raw OPEFB due to its higher surface area caused by the removal of non-cellulosic components in the cellulose. Meanwhile, the peaks at 1634 and 2891 cm^−1^ represent the –OH bending vibration of absorbed water from the strong interaction between cellulose and water [18]. A peak at 896 cm^−1^ is related to β-glycosidic linkage between the glucose rings of cellulose. [19] This indicates that the cellulose is made up of a linkage bond of saccharides that formed polysaccharides. Meanwhile, the peak that appears at 1330–1369 cm^−1^ represents the bending vibration of the C-H and C-O groups of aromatic rings in polysaccharides [20].

The disappearance of a peak at 1723 cm^−1^ in the spectrum of the treated cellulose and microcrystalline cellulose samples verifies the removal of hemicellulose and lignin. This is because the presence of lignin and hemicellulose can be detected through the appearance of those peaks due to the functional group of carbonyl ester and the C=O acetyl group of the uronic ester, respectively [4,8,21]. In addition, the peak at 1228 cm^−1^, which represents the syringyl ring unit and C-O stretching at lignin and xylan, disappeared in the spectra of the treated cellulose, suggesting the removal of lignin and hemicellulose after various treatments [15,22]. These clearly show that most non-cellulosic materials have been removed upon the alkaline treatment, bleaching and acid hydrolysis processes.

**Figure 4:**
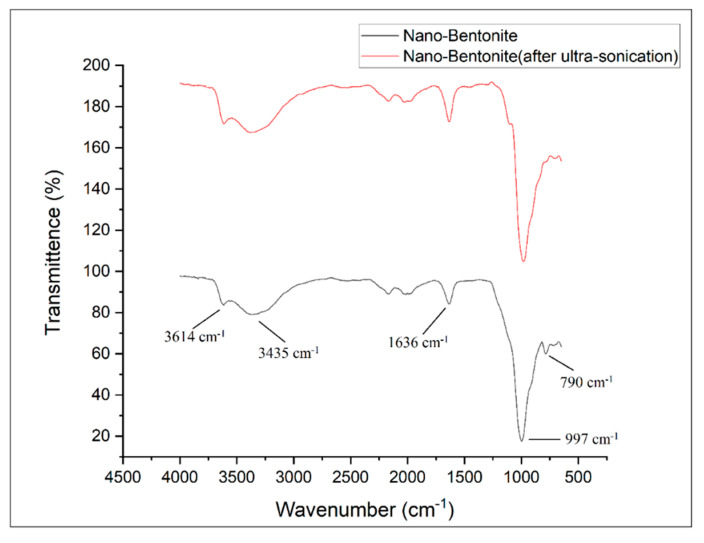
FTIR spectra of nano-bentonite (before and after ultra-sonication).

Figure 4 shows the FTIR spectra of the nano-bentonite (before and after ultra-sonication). By comparing the results of both samples, no significant change in the pattern of the FTIR spectra can be observed. This indicates that the ultra-sonication process does not alter the chemical structure of the nano-bentonite. Both samples exhibit peaks at 3614 and 3435 cm^−1^, which indicate the possibility of the hydroxyl linkage from alumino-silicate layers [23]. The peak at 3614 cm^−1^ represents the interlayer hydrogen bonding, corresponding to the stretching vibration of the OH coordinated octahedral layer of Al+Mg [18]. The bands at 1636 and 3435 cm^−1^ indicate the –OH stretching vibration due to the absorption of water in the bentonite di-octahedral surface [23]. The sharp and intense peak near 1000 cm^−^^1^ represents the Si-O-Si stretching in the nano-bentonite [24]. The peaks at 790 and 997 cm^−1^ are the characteristic signals of Al-O-Si and Si-O stretching vibrations, respectively [24]. Meanwhile, the peaks at 3614 cm^−1^, 3435 cm^−1^, 1636 cm^−1^, 997 cm^−1^ and 790 cm^−1^ imply the presence of montmorillonite (MMT) in the bentonite [23]. The ultra-sonicated nano-bentonite has a more intense peak related to Si-O stretching around 790 cm^−1^, indicating that the ultra-sonication treatment has increased the surface area of the nano-filler by delamination or the stacking platelets, which is also proved through the SEM and XRD analyses.

### 3.3. Structure of Raw OPEFB Fiber, Treated Cellulose, Microcrystalline Cellulose and Nano-Bentonite as Observed by XRD

**Figure 5:**
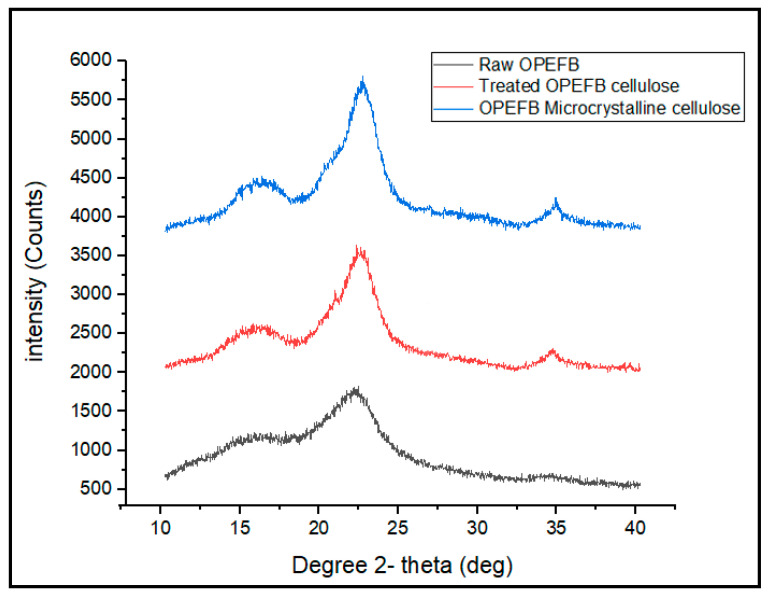
X-ray diffraction (XRD) pattern of raw OPEFB cellulose, treated cellulose and microcrystalline cellulose.

The XRD patterns of raw OPEFB fiber, treated OPEFB cellulose and OPEFB microcrystalline cellulose are shown in Figure 5. The XRD diffractograms of all the samples revealed a broad amorphous hump and crystalline peaks, indicating that OPEFB is a semi-crystalline material in nature. Generally, all samples show peaks at around 2Ɵ = 15–16°, 2Ɵ = 21–22° and 2Ɵ = 34–35°, which indicates that the OPEFB contains a crystalline structure of cellulose I with (110), (200) and (004) diffraction planes, respectively, which is typical for natural plant cellulose [25]. The lowest intensity peak at 2θ = 18° is related to amorphous region arrangement, while 2θ = 22° is related to the crystalline structure of cellulose. The CrI was calculated based on the Segal formula [26]. The raw OPEFB possesses 37.80% CrI value, which is the lowest among all samples. This can be associated with its high amorphous region. Conversely, the microcrystalline cellulose possesses the highest CrI value, which is 65.9% due to the removal of the amorphous phase from its structure.

The low Crl value of the raw OPEFB indicates that it contains a high content of hemicellulose and lignin. The same results were obtained through previous studies [27]. As expected, crystallinity of the OPEFB increased after the removal of lignin and hemicellulose. After the alkaline and bleaching pre-treatment, the peak of the treated OPEFB cellulose became sharper and more intense than the raw OPEFB, suggesting that the crystallinity of the treated OPEFB cellulose becomes higher after the treatment. However, the CrI value has been further increased when the treated OPEFB cellulose was acid hydrolyzed to form microcrystalline cellulose. This clearly indicates that more amorphous linkages within the cellulose structure have been broken down, releasing the high crystalline cellulose [28].

**Figure 6:**
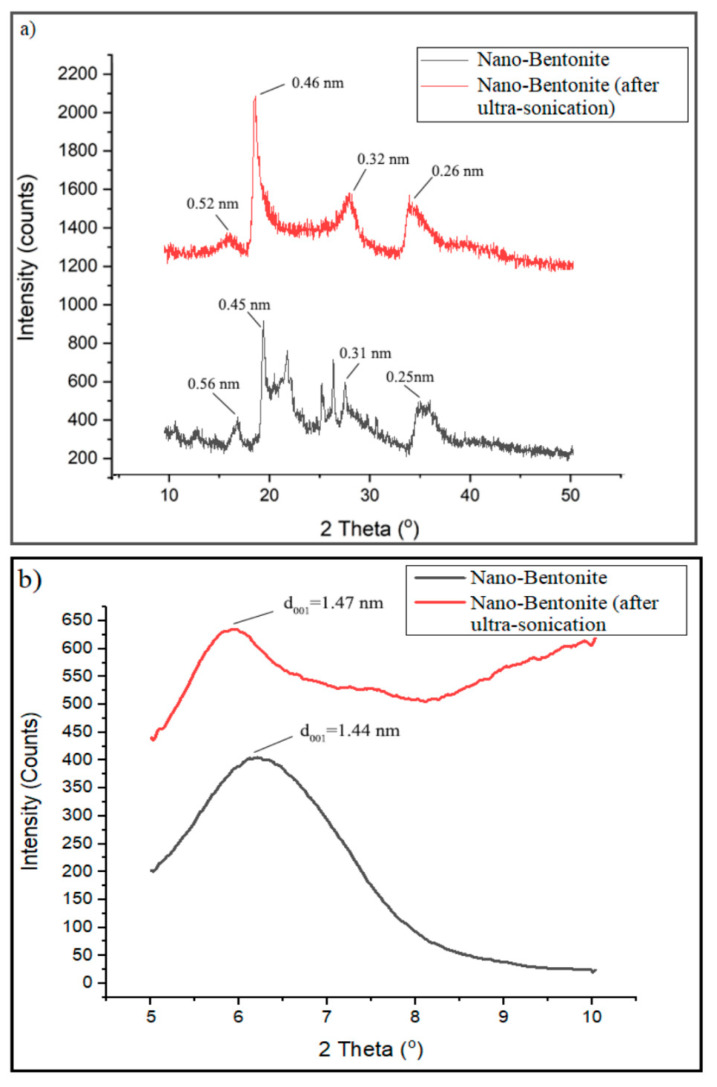
XRD pattern of nano-bentonite (before and after ultra-sonication) at 2θ = 10–50° (**a**); XRD pattern of nano-bentonite (before and after ultra-sonication) at 2θ = 5–10° (**b**).

Figure 6a displays the XRD pattern of the nano-bentonite (before and after ultra-sonication) in the region of 2Ɵ = 5° to 50°. However, the signal from 5° to 10° was focused to clearly observe the changes in d_001_ basal spacing of the nano-bentonite upon ultra-sonication (Figure 6b). The nano-bentonite’s basal spacing represents the stacking distance and order between its platelets and provides information about the platelets dispersion. Figure 6b exhibits that the d_001_ basal spacing of raw nano-bentonite is 1.44 nm. However, the d_001_ of the nano-bentonite has been increased to 1.47 nm after the ultra-sonication process. This indicates that the ultra-sonication process does slightly increase the d_001_ of the nano-bentonite, by altering the bentonite layer’s stacking through de-agglomeration of the bentonite’s tactoids. The small increase in the basal spacing of bentonite can facilitate the intercalation of the TPS chain during the mixing process and improve the film’s strength. This outcome was the same with the result of Abdul Hamid et al., where they showed that the ultra-sonication process could produce more loosely packed MMT and encourage the copolymer chains’ intercalation inter-platelets [29].

Apparently, there are clear changes in the XRD pattern of the nano-bentonite after it is subjected to the ultra-sonication process (Figure 6a). The diffraction peaks at 2θ = 21.8°, 25.4° and 26.6° disappeared, suggesting that the ultra-sonication process has successfully and effectively disrupted and at a certain level destroyed the organization of the nano-bentonite platelets [30]. Moreover, the diffraction peaks at 2θ = 19.76° and 35° are shifted to lower angles to 18.9° and 34.0°, respectively, due to the expanding of basal spacing of the nano-bentonite platelets.

### 3.4. Mechanical Analysis of TPS, TPS/B and TPS/C Bio-Composites, TPS/B/C Hybrid Bio-Composite Films

**Figure 7:**
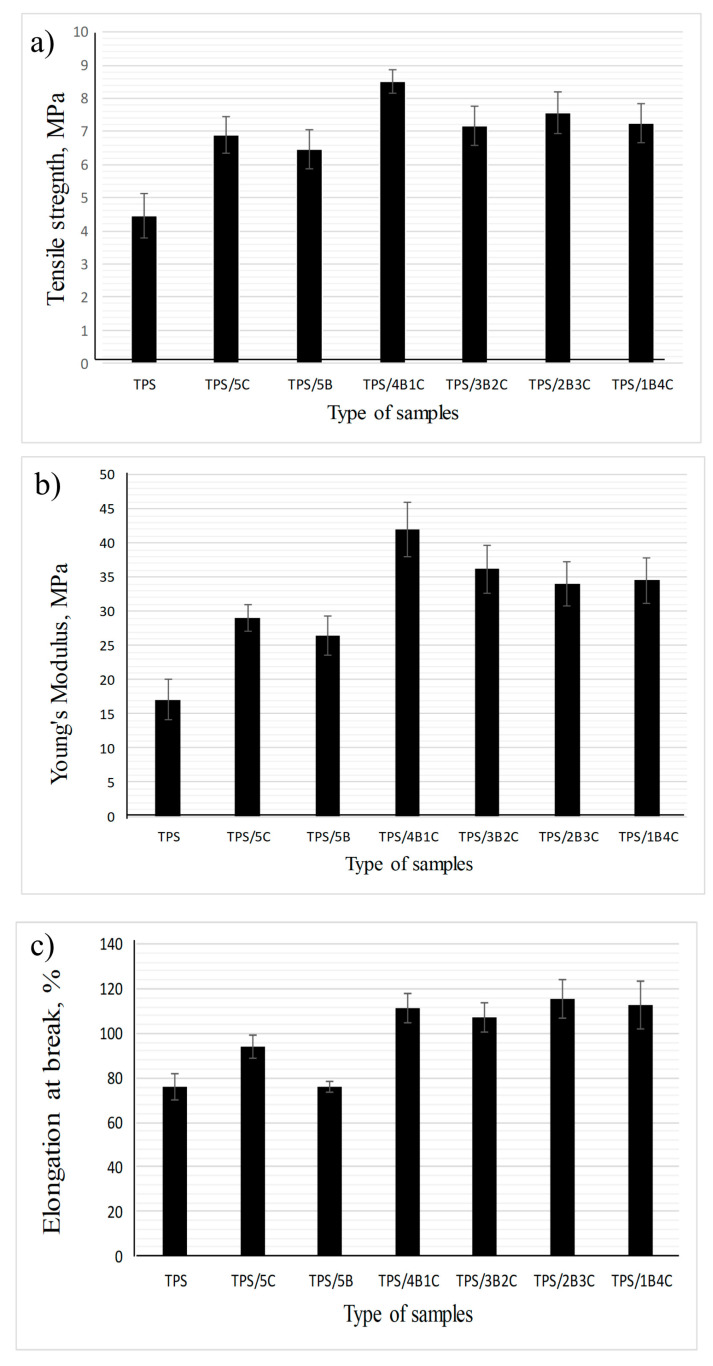
Tensile properties of TPS, TPS bio-composites and TPS hybrid bio-composite films: (**a**) tensile strength; (**b**) Young’s Modulus; (**c**) elongation at break.

Figure 7a shows the tensile strength of the TPS and TPS bio-composite film with different nano-bentonite (B) and microcrystalline cellulose (C) ratios. Regardless of the types of filler used (single or hybrid), the tensile strength value of the film increased with the incorporation of 5 wt% fillers into the TPS matrix. These results were in accordance with many reviews where phyllosilicate clay and cellulose can improve the TPS composite films’ tensile strength due to high compatibility and positive interactions such as hydrogen bonding between the TPS matrix and nanofiller, leading to better stress transfer [31,32]. The unfilled TPS exhibits the lowest tensile strength, which is 4.44 MPa, while the TPS/4B1C hybrid bio-composite film possesses the highest tensile strength (8.52 MPa), showing 92% increment when benchmarked with the unfilled TPS. This signifies that the inclusion of the hybrid B/C fillers at the optimum ratio can bring a positive synergy effect within the TPS matrix and enhance the tensile strength of the film with a more efficient load transfer mechanism. This explanation was in line with the results of Zakuwan et al., in which tensile strength improvement was obtained when cellulose nanocrystal and modified MMT was incorporated into the biopolymer. They claimed that the network forming of nanocellulose and MMT enforcement in a different dimension in the biopolymer matrix can improve bio-composite films’ reinforcement capability [33].

The results also suggest that the use of single filler (either C alone or B alone) could not allow the film to achieve higher tensile strength than the hybrid fillers 4B1C. However, the use of C resulted in a higher increment in tensile strength as compared to B. As compared to C, B tends to experience a higher degree of agglomeration in the biopolymer matrix due to its platy particles that are easily stacked together to form tactoids. Poor dispersion of tactoids reduces the efficiency of the B filler in reinforcing the matrix. C can better reinforce the TPS due to its fibrous-like particles that can transfer the load more efficiently [34]. However, when used as hybrid filler with the B, C cannot be added in more than 1 wt%. We have postulated that the high content of C in the hybrid filler system can reduce the reinforcing effect because overcrowding of the C particles may occur and inhibit good dispersion and distribution of the B’s nanoplatelets in the matrix. Poorly dispersed hybrid fillers may form a stress concentration point in the TPS matrix and reduce the tensile properties of the TPS [35,36].

The Young’s modulus values of the TPS, TPS bio-composites and TPS hybrid bio-composites with different B and C ratios are compared in Figure 7b. It can be seen that the Young’s modulus values of all the TPS bio-composites are higher than the unfilled TPS, showing that incorporating single or hybrid fillers into the TPS may improve the stiffness of the film. The unfilled TPS exhibits the lowest Young’s modulus, which is 17.1 MPa, while TPS/4B1C hybrid bio-composite demonstrates the highest Young’s modulus, which is 42.0 MPa. However, the Young’s modulus of the film has slightly decreased to 36.22 MPa when the C loading increased to 2 wt% (3B2C). Moreover, the Young’s modulus values of the TPS/2B3C and TPS/1B4C films show no significant difference with the TPS/3B2C, indicating that increases in the C loading in the matrix does not further improve the stiffness of the film. Agglomeration of C particles may occur when added in high content, reducing the matrix–filler interactions [37]. In agreement with the tensile strength data, the Young’s modulus of the TPS/5B is lower than the TPS/5C bio-composite film. As mentioned earlier, the platy particles of B can stack together in great numbers, poorly dispersed and distributed in the matrix. This reduces matrix–filler interactions that play the main role in stiffening the matrix.

Based on Figure 7c, the TPS/1B4C hybrid bio-composite film demonstrates the elongation at a break value of 117%, which is 156% higher than the unfilled TPS. This is somewhat impressive as the addition of natural filler or inorganic filler always reduces the elongation at break of the matrix. This is because filler with a stiffer characteristic reduces the flexibility of the biopolymer [9,10,11,12,13,14].

**Figure 8:**
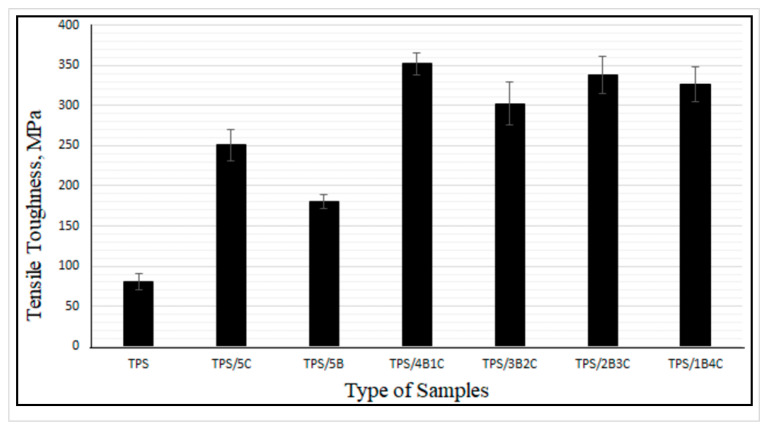
Tensile toughness of TPS, TPS bio-composites and TPS hybrid bio-composite films.

**Figure 9:**
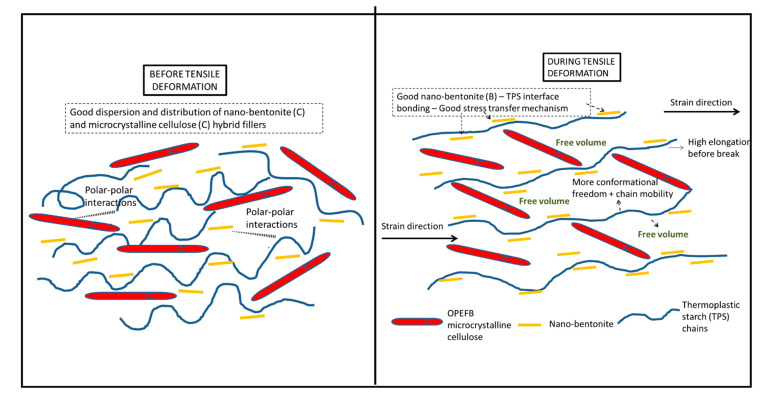
Mechanism of interactions between the TPS and the hybrid fillers during tensile deformation.

Tensile toughness is calculated based on the area under the stress–strain curve. The greater the area under the curve, the greater the toughness value will be, showing that the polymer performs greater tensile strength and elongation at break due to a more efficient energy absorption mechanism [38]. Figure 8 illustrates the tensile toughness of the TPS, TPS bio-composite and TPS hybrid bio-composite films. Overall, the results suggest that the film’s tensile toughness has been tremendously improved upon incorporation of the hybrid fillers. TPS/4B1C displays the highest value of tensile toughness with an increment of ~338%, when compared with the unfilled TPS. The difference in physical characteristics (size, shape, etc.) and chemistry of B and C fillers might contribute to a greater toughening effect on the film. We are still investigating these factors and perhaps will come out with theory and a mechanism in our next publications after finishing all the analyses. For now, we are postulating that the fibrous particles of C, when dispersed well in the matrix, not only can contribute to good stress transfer mechanism, but will also produce more free volume in the matrix when the film is stretched. Figure 9 illustrates the proposed mechanism. During tensile deformation, the small size of B nanoplatelets and the TPS molecular chains will arrange and stretch according to strain direction. This will produce greater interface bonding between the B and the TPS molecular chains, thus producing a more effective stress transferring mechanism. The bigger and longer C particles are not as mobile as the B nanoplatelets and, therefore, will not exactly follow the strain direction, but rather arrange themselves vertically between the stretched TPS molecular chains. This resulted in the formation of more free volumes in the hybrid bio-composite structure, allowing for greater starch chain mobility (entropy) and conformational freedom. The conformation of the TPS chains will stimulate chain relaxation in the stress concentrated area and allow the matrix to be more flexible and more efficient in absorbing energy through molecular motions. These interactions might responsible for contributing to the toughening effect on the film.

Comparative study was done with other TPS bio-composite systems investigated by other researchers. Based on the data obtained from recent literatures, our TPS hybrid bio-composite film’s tensile strength is on par with the values reported, while the flexibility of our film is much better than them [13,14,39]. For instance, Fazeli et al. have used cellulose fibers to reinforce the TPS derived from corn. They have applied surface modification on the cellulose fiber using the air plasma treatment to improve the matrix/fiber adhesion in the TPS bio-composite structure. The maximum tensile strength was achieved when the plasma treated cellulose fiber was added in 6 wt%. However, elongation at break value has been dropped significantly from 48.2% to 9.2% only [13]. In another work, Chen et al. have prepared TPS bio-composite films containing oxidized microcrystalline cellulose (MCC) as a reinforcing filler via a hot-compression molding technique. As compared to the original MCC, the oxidized MCC was more capable of enhancing the tensile strength of the TPS. However, the tensile strength obtained was 6.61 MPa while the elongation at break was only 40% [14]. In more recent work, Yin et al. have prepared the TPS bio-composites filled with dialdehyde lignocellulose (DLC) in the loading of 0 to 12 wt%. Maximum tensile strength was obtained when 3 wt% DLC was employed as filler with the value of 5.26 MPa. However, the bio-composite only managed to achieve elongation at a break value of 91.60% [39]. Both tensile strength and elongation at break values reported for the TPS bio-composites in the above literatures were lower than the values of our optimum hybrid bio-composite system (TPS/1B4C). As mentioned in the above discussion, the maximum tensile strength and elongation at break of the TPS hybrid bio-composite film in this study were 8.52 MPa and 117%, respectively. Apparently, the improvement in toughness value upon the inclusion of the hybrid fillers was impressive (+338%). The work by Li et al. has shown that they have successfully obtained very high tensile strength starch-based bio-composites when using montmorillonite and cellulose nanofiber as hybrid fillers. However, the elongation at break values of the samples was very low (less than 7%) compared to our hybrid TPS bio-composite films (117%). The above findings proved that that the origin of starch, method of processing and types of filler/hybrid fillers used will determine the obtained tensile strength and elongation at break of the bio-composite film. These are in agreement with review papers wrote by Pérez-Pacheco et al. [11] and Xie et al. [31]. In our case, the unfilled TPS has shown very moderate tensile strength, but the hybrid B/C fillers have played their role in simultaneously enhancing the tensile strength and elongation at break of the TPS matrix. Thus, significant enhancement in the tensile toughness of the film could be observed. This trend is rarely obtained and reported. Nevertheless, we believe that our films that possess good flexibility and toughness will suit for certain applications such as for short term flexible packaging and wrapping plastic.

### 3.5. The Interactions of Single and Hybrid Fillers with the TPS Matrix as Observed through FTIR and XRD Analyses

**Figure 10:**
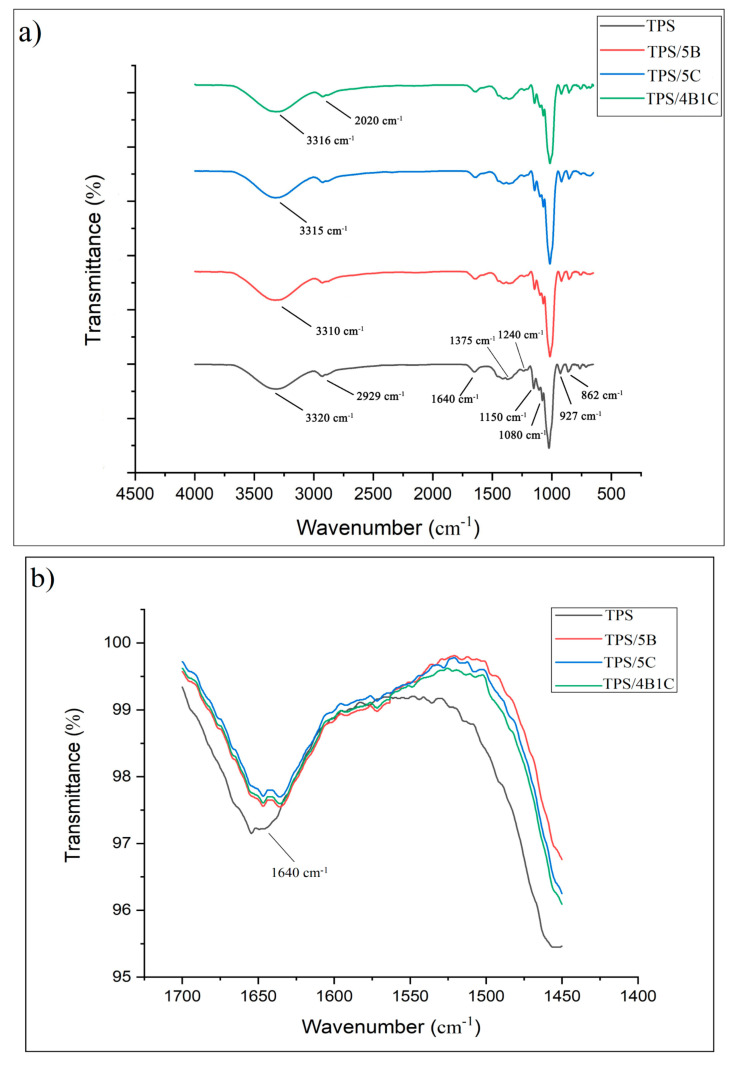
FTIR of unfilled TPS, TPS bio-composites and TPS hybrid bio-composite, in the region of (**a**) 650–4000 cm^−1^ and (**b**) 1450–1700 cm^−1^.

FTIR analysis was used to interpret the chemical functionalities’ presence in the bio-composite’s chemical structure and analyze a potential interaction between the TPS matrix and single filler and with the hybrid filler system. FTIR is sensitive to detecting the change of TPS structure at the molecular level, such as chain conformation, crystallinity, water content, and TPS and filler interaction [40]. According to Figure 10, the FTIR spectra of all the samples indicate a broad band located at 3000 to 3700 cm^−1^, corresponding to free, inter-and intramolecular O-H stretching. This indicates the presence of a high amount of O-H functional group of the TPS and TPS bio-composite films’ structure [41]. For the unfilled TPS film, wavelength from 800 to 1200 cm^−1^ is the fingerprint region of the TPS, contributing to glucan ring vibration by C-OH stretching and bending vibration and the C-O-C glycoside bond vibration [42]. A single peak observed at 1640 cm^−1^ represents the water—bound tightly in TPS film due to its hygroscopic nature [42].

The characteristic peak at 2929 cm^−1^ was attributed to asymmetrical C-H stretching and vibration. Meanwhile, the characteristic peak at 1375 cm^−1^ represents the –CH_2_ bending. Furthermore, there is a small peak at 1153 and 1080 cm^−1^ representing the C-O stretch in the C-O-H group in the TPS film, whereas 1240 cm^−1^ shows the C-O stretch of C-O-C bond in the structure of the film [41,43]. The peaks at 927 and 862 cm^−1^ represent the starch glycosidic linkage of glucose in starch [41]. Overall, the FTIR spectra for all the TPS bio-composite films are almost similar to the unfilled TPS films but there was a slight change in the intensity of some peaks. Furthermore, the shifting of certain bands was also noticed.

In the region of 3000 to 3700 cm^−1^, the band appears due to O-H stretching that provides information related to hydrogen bonding between TPS and fillers. For TPS/5B bio-composite, the disappearance of the nano-bentonite peak at 3435 cm^−1^ is associated with the hydroxyl linkage formation within the alumino-silicate layered structure of the clay, indicating that the filler is forming new hydrogen bonding with the TPS. XRD analysis has further proved this by showing a broadening of d_001_ peak for the nano-bentonite. For the TPS/5B sample, there is a shift of peak at 3320 cm^−1^ to a lower wavenumber, which is 3310 cm^−1^, indicating new and stable hydrogen bonds formed in the TPS bio-composite films [44]. Hydrogen bonding has been developed due to compatibility between the TPS and B filler.

Previous research showed that there is good interfacial bonding between the cellulose and TPS due to their chemical similarity and good compatibility that allow for the formation of hydrogen bonding between them [45]. Based on the FTIR spectra of TPS/5C, it can be observed that incorporation of C into the TPS matrix has slightly sharpened the peak and also shifted the peak to a lower wavenumber (3315 cm^−1^). This can be associated with the O-H vibration of the high crystalline structure of the C filler. This outcome was in accordance with the study of Zhang et al. They have concluded that the O-H stretching vibration shifts to a lower wavenumber in the FTIR spectrum due to new hydrogen bonding between TPS and nanocellulose [46]. For the analysis of the TPS/4B1C hybrid bio-composite film, interactions between the TPS and hybrid fillers can also be realized through the FTIR data. Since both of the single fillers showed interaction with TPS films by forming hydrogen bonding, hybrid fillers are expected to interact with TPS films in the same ways. Peaks at 2929 (C-H) and 3320 cm^−1^ of the TPS were shifted to 2020 and 3316 cm^−1^ due to new hydrogen forming between the TPS and hybrid fillers (B and C). This indicates that hybrid fillers have good compatibility with the TPS matrix by forming strong polar hydroxyl interactions.

**Figure 11:**
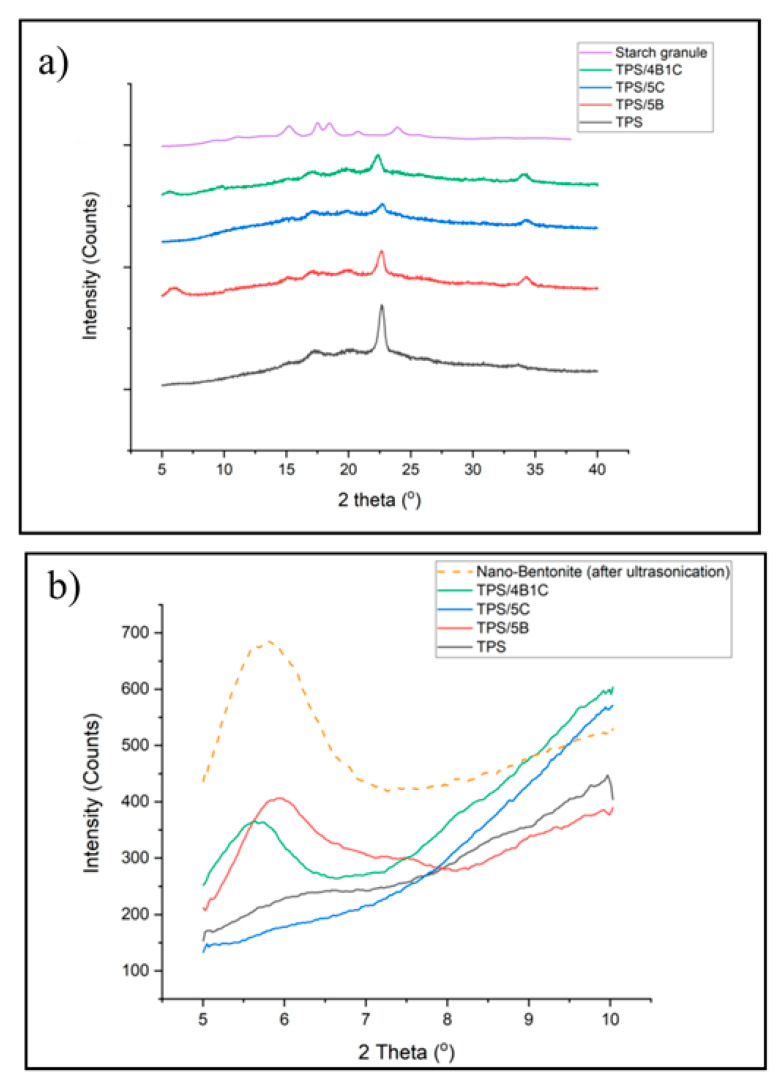
XRD diagram of TPS, TPS bio-composite and TPS hybrid bio-composite films, at the region of (**a**) 2θ = 5–40° and (**b**) 2θ = 5–10°.

Figure 11a shows the XRD pattern of the unfilled TPS, TPS bio-composite and TPS hybrid bio-composite films. TPS has experienced a reduction in peak intensity in the region of 2Ɵ = 10–40° when added with filler (either single or hybrid fillers) due to the reduction in retrogradation rate that occurs in the matrix. Interface interactions between the matrix and fillers can slow down the retrogradation process of the starch. During the TPS matrix cooling, the amylose and amylopectin chains are starting to arrange back into an ordered structure different from native starch granules. The retrogradation process involved a few steps: extrusion of water, an increase in viscosity, gel formation and forming a crystalline structure. The typical retrogradation peaks of TPS can be seen at 17° and 22.6°.

The XRD pattern of the TPS/5C film presents the typical peak of retrograded starch structure (type B and type Vh peak) and the signal of the C filler. However, due to the similar chemical structure of TPS and microcrystalline cellulose in the TPS/5C composite, the XRD pattern shows superimposition of both parent components balanced by the composition. This is in line with the study of Dufresne et al. [37]. Incorporation of C into the TPS has brought a reduction in the intensity of Vh peaks in the XRD spectrum. This was possibly due to the transcrystallization of amylose and amylopectin on the microcrystalline cellulose surface, leading to a reduction of the starch chain’s recrystallization. The same observation was reported in the study of Fourati et al. They found that the Vh-type structure of the TPS reduced with the increase in the cellulose nanofiber content [47].

It can also be observed that the intensity of the peak related to the Vh-type structure of the TPS is lower in the TPS/4B1C hybrid bio-composite as compared to the unfilled TPS. The Vh-type structure formed in TPS was due to amylose’s recrystallization with plasticizer in the helix channel. Reduction in the peak intensity of the Vh type crystalline structure indicates that the retrogradation of the TPS was hindered with the incorporation of the single B filler or hybrid B/C fillers. The anti-retrogradation of the TPS films with the nano-bentonite was due to the hydrophilicity of bentonite clay’s surface that increased the interaction between the starch chain and bentonite platelets, affecting the dynamic rearrangement of the starch chain. Consequently, the ability of the TPS chains to recrystallize was reduced. This trend was also reported by Lara et al., where they found that the retrogradation of starch plasticized by water and glycerol was reduced by incorporating MMT [48]. Interestingly, the TPS/4B1C hybrid bio-composite film exhibits a smaller and broader peak of the Vh-type structure as compared to the TPS/5B bio-composite. This proved that the use of hybrid filler with a low content of C (1 wt%) may result in greater efficiency in preventing retrogradation than the use of single C or single B filler.

Next, the XRD signal from 5° to 10° was focused to have a clear comparison on the d_001_ basal spacing of the nano-bentonite clay before and after being incorporated into the TPS matrix. As expected, there is no peak that can be observed in the XRD signal of the unfilled TPS and TPS/5C bio-composite in the low-angle region (2Ɵ = 5–10°) since TPS is amorphous. It is known that the peak that appears at a low angle value between 5–10° represents the d_001_ basal spacing of bentonite clay [8]. When single B filler or hybrid B/C fillers were incorporated into the TPS, the d_001_ peak of B had shifted to a lower angle, indicating an increase in the interlayer basal spacing of the clay due to the intercalation of TPS chains and microcrystalline cellulose into the layered silicate structure of bentonite without complete exfoliation.

The TPS/4B1C hybrid bio-composite film shows the highest increment in basal spacing in which the d_001_ increased from 1.47 to 1.55 nm. Moreover, broadening of the d_001_ peak can clearly be seen. It seems that the inter-platelets of the nano-bentonite have been well intercalated by the TPS chains with the presence of a small amount of the microcrystalline cellulose. This could be due to the capability of bentonite to interact with the C filler, other than the host biopolymer. As mentioned earlier, there are strong polar interactions between the TPS, C and B fillers due to the hydroxyl group composition within their structure. The interface interactions between B and C help to pull the B platelets away from its tactoid structure, making a wider space for the TPS chains intercalation. This factor explains why the TPS/4B1C performed the greatest mechanical properties compared to other TPS bio-composite films.

### 3.6. Fractured Surface Morphology of the TPS Bio-Composite and TPS Hybrid Bio-Composite Films as Observed through Scanning Electron Microscopy (SEM) Analyses

**Figure 12:**
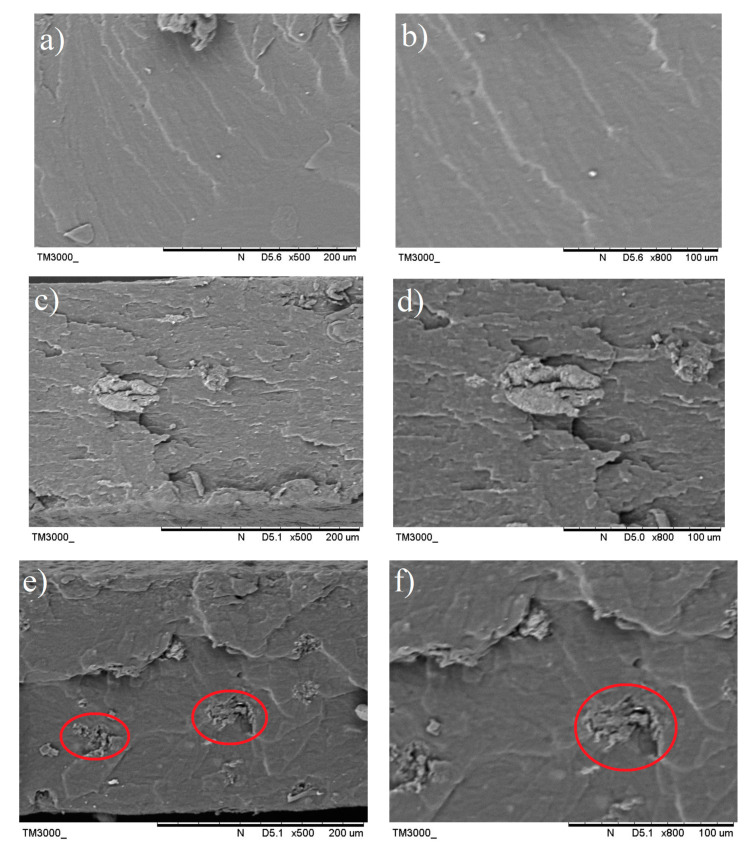

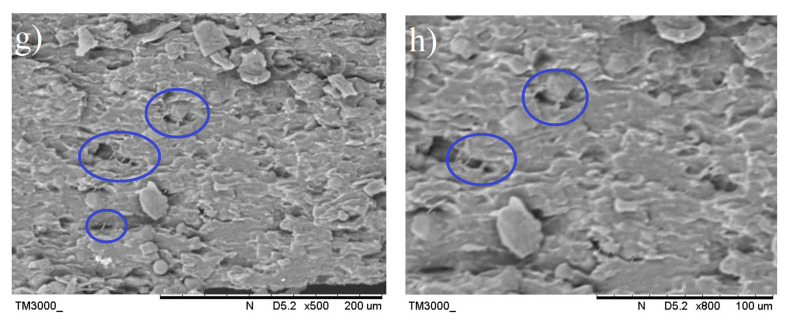
SEM images of the tensile fractured surface of the (**a**,**b**) TPS films, (**c**,**d**) TPS/5B, (**e**,**f**) TPS/5C, (**g**,**h**) and TPS/4B1C at 500× magnification (**left**) and 800× magnification (**right**)**.**

The tensile fractured surface morphologies of the TPS, TPS bio-composite and TPS hybrid bio-composite were studied by Scanning Electron Microscopy (SEM). Figure 12a shows that the fractured surface of the TPS films was uniform and no residual for the starch granule structure can be observed. The smooth surface indicates that the origin starch granule structure was completely disrupted and broken when starch was plasticized with glycerol and water at a high temperature. The TPS film possesses a smooth surface showing that the unfilled TPS underwent a very low degree of matrix deformation and easily fracture upon the application of a small tensile load/force. Both TPS/5C (Figure 12e) and TPS/5B (Figure 12b) also show a clean and smooth fracture surface, indicating that both types of the TPS bio-composites have experienced some degree of matrix deformation upon tensile loading, but not much. For the TPS/5B bio-composite film, no image of B filler could be captured due to the very small size of the nano-bentonite. However, for the TPS/5C bio-composite film, the fractured surface shows some aggregation of C (circle in red color) inside the matrix of the TPS due to the strong intramolecular hydrogen bonding between the particles of C. This filler–filler interaction caused aggregation of C in the TPS matrix and prohibited the bio-composite to achieve a higher tensile strength. This was in line with the study of Zhang et al. where the nanocellulose agglomeration phenomenon in the host TPS had caused uneven loading distribution, void formation in the matrix and subsequently led to premature failure [33]. Small voids were observed due to the agglomerated C fiber pullout from the matrix phase. The C fiber was pulled out in the same direction of the tensile force applied. By adding 1% of C as co-filler with B, the fractured surface morphology of the TPS film has changed drastically. The surface roughness increased due to the occurrence of significant matrix deformation. This tallies with the tensile data which indicate that the elongation at break of this TPS/4B1C film was higher than the unfilled TPS and TPS bio-composite with single filler. However, there is no agglomeration and aggregation of C spotted in the SEM images of the TPS/4B1C, indicating that the C particles have been well dispersed inside the TPS matrix due to the good compatibility and interactions with the matrix and B filler. Furthermore, there is no fiber pullout that can be observed in the surface of the TPS/4B1C bio-composite film. This could be attributed to better interface interaction between C and the matrix with the presence of B. The C fiber was well wetted and embedded in the TPS matrix and led to high contact surface area between C and the TPS. In addition, the appearance of a tiny fibril structure bridging the small voids (blue color circle in Figure 12g,h) was noticed, suggesting that the C filler has been vertically oriented to the force applied during the tensile test. A similar structure of nanocellulose bridging in TPS was also observed by Li et al. [40].

## 4. Conclusions

Microcrystalline cellulose (C) was extracted from the raw OPEFB fiber and nano-bentonite (B) was ultra-sonicated to reduce the large tactoid structure. The changes in structure and morphology of the fillers from their original form after being subjected to chemical treatment and the ultra-sonication process have been witnessed and confirmed through FTIR, XRD and SEM analyses. Both fillers were used to form hybrid fillers in the production of the TPS hybrid bio-composite film. The TPS bio-composite films and TPS hybrid bio-composite films were produced by the casting method and the effects of the OPEFB microcrystalline cellulose (C)/nano-bentonite (B) ratio on the mechanical properties of the resultant TPS bio-composite films were studied. The highest tensile strength, Young’s modulus and tensile toughness was achieved when the TPS was incorporated with 4 wt% B and 1% of C filler (TPS/4B1C). The use of this hybrid filler system resulted in a more significant enhancement in the tensile properties of the film as compared to the single B or C filler. The results signify that B/C hybrid fillers have brought a positive synergistic effect to the TPS matrix. The good compatibility between C, B and TPS was observed through the FTIR, XRD and SEM analyses. The findings demonstrate that the combination of the OPEFB microcrystalline cellulose and nano-bentonite can produce an efficient hybrid filler system that allows for greater enhancement in the mechanical properties of the TPS-based film for packaging applications. Our work signifies that this hybridization approach is a powerful method to toughen the TPS film. This encourages further exploration on the potential of this hybrid filler system for toughening other bio-polymers/polymers. More importantly, all the ingredients used to form the hybrid TPS bio-composite are environmentally friendly and renewable, thus contributing to the development of sustainable packaging materials.

## Figures and Tables

**Table 1:** The formulation of thermoplastic (TPS) bio-composites and TPS hybrid bio-composites containing hybrid filler (C/B).

TPS (wt%)	Microcrystalline Cellulose (C) (wt%)	Nano-Bentonite (B) (wt%)	Acronym
100	-	-	TPS
95	5	-	TPS/5C *
95	1	4	TPS/4B1C^+^
95	2	3	TPS/3B2C ^+^
95	3	2	TPS/2B3C ^+^
95	4	1	TPS/1B4C ^+^
95	5	-	TPS/5B *

* TPS bio-composite ^+^ TPS hybrid bio-composite.

## Data Availability

The data presented in this study are available on request from the corresponding author.
